# Comparison of linear discriminant analysis methods for the classification of cancer based on gene expression data

**DOI:** 10.1186/1756-9966-28-149

**Published:** 2009-12-10

**Authors:** Desheng Huang, Yu Quan, Miao He, Baosen Zhou

**Affiliations:** 1 Department of Mathematics, College of Basic Medical Sciences, China Medical University, Shenyang 110001, China; 2 Key Laboratory of Cancer Etiology and Intervention, University of Liaoning Province, China; 3 Computer Center, Affiliated Shengjing Hospital, China Medical University, Shenyang 110004, China; 4 Information Center, the First Affiliated Hospital, China Medical University, Shenyang 110001, China; 5 Department of Epidemiology, School of Public Health, China Medical University, Shenyang 110001, China

## Abstract

**Background:**

More studies based on gene expression data have been reported in great detail, however, one major challenge for the methodologists is the choice of classification methods. The main purpose of this research was to compare the performance of linear discriminant analysis (LDA) and its modification methods for the classification of cancer based on gene expression data.

**Methods:**

The classification performance of linear discriminant analysis (LDA) and its modification methods was evaluated by applying these methods to six public cancer gene expression datasets. These methods included linear discriminant analysis (LDA), prediction analysis for microarrays (PAM), shrinkage centroid regularized discriminant analysis (SCRDA), shrinkage linear discriminant analysis (SLDA) and shrinkage diagonal discriminant analysis (SDDA). The procedures were performed by software R 2.80.

**Results:**

PAM picked out fewer feature genes than other methods from most datasets except from Brain dataset. For the two methods of shrinkage discriminant analysis, SLDA selected more genes than SDDA from most datasets except from 2-class lung cancer dataset. When comparing SLDA with SCRDA, SLDA selected more genes than SCRDA from 2-class lung cancer, SRBCT and Brain dataset, the result was opposite for the rest datasets. The average test error of LDA modification methods was lower than LDA method.

**Conclusions:**

The classification performance of LDA modification methods was superior to that of traditional LDA with respect to the average error and there was no significant difference between theses modification methods.

## Background

Conventional diagnosis of cancer has been based on the examination of the morphological appearance of stained tissue specimens in the light microscope, which is subjective and depends on highly trained pathologists. Thus, the diagnostic problems may occur due to inter-observer variability. Microarrays offer the hope that cancer classification can be objective and accurate. DNA microarrays measure thousands to millions of gene expressions at the same time, which could provide the clinicians with the information to choose the most appropriate forms of treatment.

Studies on the diagnosis of cancer based on gene expression data have been reported in great detail, however, one major challenge for the methodologists is the choice of classification methods. Proposals to solve this problem have utilized many innovations including the introduction of sophisticated algorithms for support vector machines [1] and the proposal of ensemble methods such as random forests [2]. The conceptually simple approach of linear discriminant analysis (LDA) and its sibling, diagonal discriminant analysis (DDA) [3-5], remain among the most effective procedures also in the domain of high-dimensional prediction. In the present study, our main focus will be solely put on the LDA part and henceforth the term "discriminant analysis" will stand for the meaning of LDA unless otherwise emphasized. The traditional way of doing discriminant analysis is introduced by R. Fisher, known as the linear discriminant analysis (LDA). Recently some modification of LDA have been advanced and gotten good performance, such as prediction analysis for microarrays (PAM), shrinkage centroid regularized discriminant analysis(SCRDA), shrinkage linear discriminant analysis(SLDA) and shrinkage diagonal discriminant analysis(SDDA). So, the main purpose of this research was to describe the performance of LDA and its modification methods for the classification of cancer based on gene expression data.

Cancer is not a single disease, there are many different kinds of cancer, arising in different organs and tissues through the accumulated mutation of multiple genes. Many previous studies only focused on one method or single dataset and gene selection is much more difficult in multi-class situations [6,7]. Evaluation of the most commonly employed methods may give more accurate results if it is based on the collection of multiple databases from the statistical point of view.

In summary, we investigate the performance of LDA and its modification methods for the classification of cancer based on multiple gene expression datasets.

## Methods

**Figure 1:**
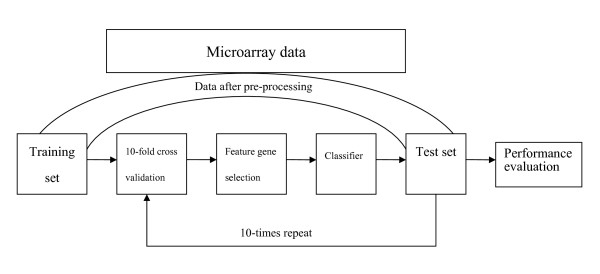
**Framework for the procedure of classification**.

Procedure for the classification of cancer is shown as follows. First, a classifier is trained on a subset (training set) of gene expression dataset. Then, the mature classifier is used for unknown subset (test set) and predicting each observation's class. The detailed information about classification procedure is shown in Figure 1.

### Datasets

Six publicly available microarray datasets [8-14] were used to test the above described methods and we call them 2-class lung cancer, colon, prostate, multi-class lung cancer, SRBCT and brain following the naming there. Due to the fact that microarray-based studies may report findings that are not reproducible, after reviewing literature we selected these above public datasets with the consideration of our research topic and cross-comparison with other similar studies. The main features of these datasets are summarized in Table 1.

**Table 1:** Characteristics of the six microarray datasets used

Dataset	No. of samples	Classes (No. of samples)	No. of genes	**Original ref**.	Website
Two-class lung cancer	181	MPM(31), adenocarcinoma(150)	12533	[8]	http://www.chestsurg.org
Colon	62	normal(22), tumor(40)	2000	[9]	http://microarray.princeton.edu/oncology/affydata/index.html
Prostate	102	normal(50), tumor(52)	6033	[10]	http://microarray.princeton.edu/oncology/affydata/index.html
Multi-class lung cancer	68(66) ^a^	adenocarcinoma(37), combined(1), normal(5), small cell(4), squamous cell(10), fetal(1), large cell(4), lymph node(6)	3171	[11,12]	http://www-genome.wi.mit.edu/mpr/lung/
SRBCT	88(83) ^b^	Burkitt lymphoma (29), Ewing sarcoma (11), neuroblastoma (18), rhabdomyosarcoma (25), non-SRBCTs(5)	2308	[13]	http://research.nhgri.nih.gov/microarray/Supplement/
Brain	42(38) ^c^	medulloblastomas(10), CNS AT/RTs(5), rhabdoid renal and extrarenal rhabdoid tumours(5), supratentorial PNETs(8), non-embryonal brain tumours (malignant glioma) (10), normal human cerebella(4)	5597	[14]	http://research.nhgri.nih.gov/microarray/Supplement/

Note: Some samples were removed for keeping adequate number of each type.

a. One combined and one fetal cancer samples were removed, and real sample size is 66;

b. Five non-SRBCT samples were removed, and real sample size is 83;

c. Four normal tissue samples were removed, and real sample size is 38.

### Data pre-processing

To avoid the noise of the dataset, pre-processing was necessary in the analysis. Absolute transformation was first performed on the original data. The data was transformed to have a mean of 0 and standard deviation of 1 after logarithmic transformation and normalization. When the original data had already experienced the above transformation, it entered next step directly.

### Algorithms for feature gene selection

#### Notation

Let x_ij _be the expression level of gene j in the sample i, and y_i _be the cancer type for sample i, j = 1,...,p and response y_i_∈{1,...,K}. Denote Y = (y_1_,...,y_n_)^T ^and x_i _= (x_i1_,...,x_ip_)^T^, i = 1,...,n. Gene expression data on p genes for n mRNA samples may be summarized by an n × p matrix X = (x_ij_)_n × p_. Let C_k _be indices of the n_k _samples in class k, where n_k _denotes the number of observations belonging to class k, n = n_1_+...+n_K_. A predictor or classifier for K tumor classes can be built from a learning set L by C(.,L); the predicted class for an observation x* is C(x*,L). The jth component of the centroid for class k is , the jth component of the overall centroid is .

#### Prediction analysis for microarrays/nearest shrunken centroid method, PAM/NSC

PAM [3] algorithm tries to shrink the class centroids () towards the overall centroid .(1)

where d_kj _is a t statistic for gene j, comparing class k to the overall centroid, and s_j _is the pooled within-class standard deviation for gene j:(2)

and , s_0 _is a positive constant and usually equal to the median value of the s_j _over the set of genes.

Equation(1) can be transformed to(3)

PAM method shrinks each d_kj _toward zero, and giving  yielding shrunken centroids(4)

Soft thresholding is defined by(5)

where + means positive part (t_+ _= t if t>0 and zero otherwise). For a gene j, if d_kj _is shrunken to zero for all classes k, then the centroid for gene j is , the same for all classes. Thus gene j does not contribute to the nearest-centroid computation. Soft threshold Δ was chosen by cross-validation.

#### Shrinkage discriminant analysis, SDA

In SDA, Feature selection is controlled using higher criticism threshold (HCT) or false non-discovery rates (FNDR) [5]. The HCT is the order statistic of the Z-score corresponding to index *i *maximizing , π_i _is the p-value associated with the ith Z-score and π_(i) _is the *i*th order statistic of the collection of p-values(1 ≤ *i *≤ p). The ideal threshold optimizes the classification error. SDA consists of Shrinkage linear discriminant analysis (SLDA) and Shrinkage diagonal discriminant analysis (SDDA) [15,16].

#### Shrunken centroids regularized discriminant analysis, SCRDA

There are two parameters in SCRDA [4], one is α (0<α<1), the other is soft threshold Δ. The choosing the optimal tuning parameter pairs (α, Δ) is based on cross-validation. A "Min-Min" rule was followed to identify the optimal parameter pair (α, Δ):

First, all the pairs (α, Δ) that corresponded to the minimal cross-validation error from training samples were found.

Second, the pair or pairs that used the minimal number of genes were selected.

When there was more than one optimal pair, the average test error based on all the pairs chosen would be calculated. As traditional LDA is not suitable to deal with the "large *p*, small *N*" paradigm, so we did not adopt it to select feature genes.

### Algorithms of LDA and its modification methods for classification

#### Linear discriminant analysis, LDA

Fisher linear discriminant analysis (FLDA, or for short, LDA) [17] projects high dimension data x into one dimension axle to find linear combinations **xa **with large ratios of between-group to within-group sums of squares. Fisher's criteria can be defined as:(6)

Where *B *and *W *denote the matrices of between-group and within-group sums of squares and cross-products.

Class *k *sample means  can be gotten from learning set L, and for a new tumor sample with gene expression x*, the predicted class for x* is the class whose mean vector  is closest to x* in the space of discriminant variables, that is(7)

where , v_*l *_is eigenvector, *s *is the number of feature genes.

When numbers of classes K = 2, FLDA yields the same classifier as the maximum likelihood (ML) discriminant rule for multivariate normal class densities with the same covariance matrix.

#### Prediction analysis for microarrays/nearest shrunken centroid method, PAM/NSC

PAM [3] assumes that genes are independent, the target classes correspond to individual (single) clusters and classify test samples to the nearest shrunken centroid, again standardizing by s_j _+s_0_. The relative number of samples in each class is corrected at the same time. For a test sample (a vector) with expression levels x*, the discriminant score for class k was defined by,(8)

where π_k _= n_k_/n or π_k _= 1/K is class prior probability, . This prior probability gives the overall frequency of class k in the population. The classification rule is(9)

Here  was the diagonal matrix taking the diagonal elements of . If the smallest distances are close and hence ambiguous, the prior correction gives a preference for larger classes, because they potentially account for more errors.

#### Shrinkage discriminant analysis, SDA

The corresponding discriminant score [5] was defined by(10)

Where , *P *= (*ρ*_ij_) and 

#### Algorithm of SCRDA

A new test sample was classified by regularized discriminant function [4],(11)

Covariance was estimated by(12)

where 0 ≤ *α *≤ 1

In the same way, sample correlation matrix  was substituted by .

Then the regularized sample covariance matrix was computed by 

### Study design and program realization

We used 10-fold cross-validation (CV) to divide the pre-processed dataset into 10 approximately equal-size parts by random sampling. It worked as follows: we fit the model on 90% of the samples and then predicted the class labels of the remaining 10% (the test samples). This procedure was repeated 10 times to avoid overlapping test sets, with each part playing the role of the test samples and the errors on all 10 parts added together to compute the overall error [18]. R software (version 2.80) with packages MASS, pamr, RDA, SDA was used for the realization of the above described methods [19]. A tolerance value was set to decide if a matrix is singular. If variable had within-group variance less than tol^2, LDA fitting iteration would stop and report the variable as constant. In practice, we set a very small tolerance value 1 × 10^-14^, and no singular was detected.

## Results

### Feature genes selection

As shown in Table 2, PAM picked out fewer feature genes than other methods from most datasets except from Brain dataset. For the two methods of shrinkage discriminant analysis, SLDA selected more genes than SDDA from most datasets except from 2-class lung cancer dataset. When comparing SLDA with SCRDA, SLDA selected more genes than SCRDA from 2-class lung cancer, SRBCT and Brain dataset, the result was opposite for the rest datasets.

**Table 2:** Numbers of feature genes selected by 4 methods for each dataset

Dataset	PAM	SDDA	SLDA	SCRDA
2-class lung cancer	7.98	422.74	407.83	118.72
Colon	25.72	65.67	117.08	214.87
Prostate	83.13	120.53	187.91	217.47
Multi-class lung cancer	45.26	57.98	97.27	1015.00
SRBCT	30.87	114.32	131.24	86.22
Brain	69.11	115.04	182.01	26.83

### Performance comparison for methods based on different datasets

The performance of the methods described above was compared by average test error using 10-fold cross validation. We ran 10 cycles of 10-fold cross validation. The average test errors were calculated based on the incorrectness of the classification of each testing samples. For example, for the 2-class lung cancer dataset, using the LDA method based on PAM as the feature gene method, 30 samples out of 100 sample test sets were incorrectly classified, resulting in an average test error of 0.30.

**Figure 2:**
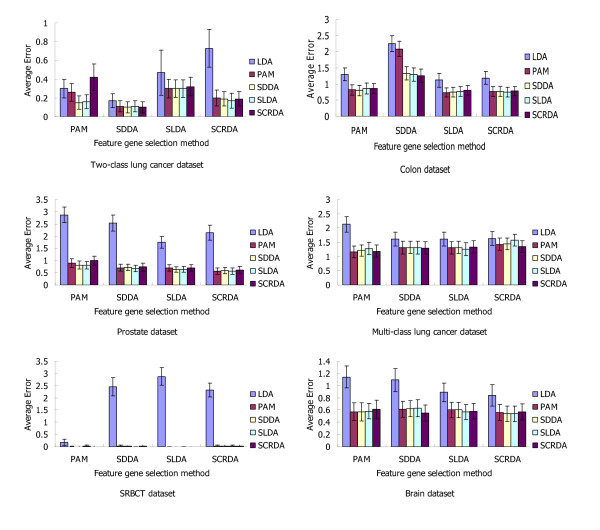
**Comparison of classification performance for different datasets**. The y-axis shows the average error and the x-axis indicates the gene selection methods: PAM, SDDA, SLDA and SCRDA. Error bars (± 1.96 SE) are provided for the classification methods.

The significance of the performance difference between these methods was judged depending on whether or not their 95% confidence intervals of accuracy overlapped. Here, if the upper limit was greater than 100%, it was treated as 100%. If two methods had non-overlapping confidence intervals, their performances were significantly different. The bold fonts in Table 3 shows the performances of PAM, SDDA, SLDA and SCRDA, when they were used both for feature gene selection and classification. As shown in Table 3, the performance of LDA modification methods is superior to traditional LDA method, while there is no significant difference between theses modification methods (Figure 2).

**Table 3:** Average test error of LDA and its modification methods (10 cycles of 10-fold cross validation)

Dataset	Gene selection methods	Performance				
		LDA	PAM	SDDA	SLDA	SCRDA
2-class Lung cancer data(n = 181, p = 12533, K = 2)	PAM	0.30	**0.26**	0.15	0.16	0.42
	SDDA	0.17	0.11	**0.1**	0.11	0.1
	SLDA	0.47	0.3	0.3	**0.3**	0.32
	SCRDA	0.73	0.20	0.19	0.17	**0.19**
						
Colon data(n = 62, p = 2000, K = 2)	PAM	1.30	**0.82**	0.8	0.86	0.86
	SDDA	2.25	2.09	**1.33**	1.29	1.25
	SLDA	1.12	0.74	0.75	**0.77**	0.80
	SCRDA	1.19	0.77	0.77	0.75	**0.78**
						
Prostate data(n = 102, p = 6033, K = 2)	PAM	2.87	**0.89**	0.82	0.81	1.00
	SDDA	2.53	0.71	**0.72**	0.68	0.74
	SLDA	1.75	0.7	0.64	**0.64**	0.70
	SCRDA	2.15	0.57	0.59	0.57	**0.61**
						
Multi-class lung cancer data(n = 66, p = 3171, K = 6)	PAM	2.13	**1.16**	1.21	1.28	1.19
	SDDA	1.62	1.32	**1.32**	1.31	1.30
	SLDA	1.62	1.31	1.32	**1.26**	1.34
	SCRDA	1.63	1.43	1.45	1.58	**1.35**
						
SRBCT data(n = 83, p = 2308, K = 4)	PAM	0.17	**0.01**	0.01	0.03	0.01
	SDDA	2.45	0.03	**0.02**	0	0.03
	SLDA	2.87	0	0	**0**	0
	SCRDA	2.32	0.03	0.03	0.02	**0.03**
						
Brain data(n = 38, p = 5597, K = 4)	PAM	1.14	**0.57**	0.57	0.58	0.61
	SDDA	1.09	0.61	**0.62**	0.63	0.55
	SLDA	0.89	0.60	0.60	**0.57**	0.58
	SCRDA	0.84	0.56	0.54	0.54	**0.57**

## Discussion

Microarrays are capable of determining the expression levels of thousands of genes simultaneously and hold great promise to facilitate the discovery of new biological knowledge [20]. One feature of microarray data is that the number of variables *p *(genes) far exceeds the number of samples *N*. In statistical terms, it is called 'large *p*, small *N*' problem. Standard statistical methods in classification do not work well or even at all, so improvement or modification of existing statistical methods is needed to prevent over-fitting and produce more reliable estimations. Some ad-hoc shrinkage methods have been proposed to utilize the shrinkage ideas and prove to be useful in empirical studies [21-23]. Distinguishing normal samples from tumor samples is essential for successful diagnosis or treatment of cancer. And, another important problem is in characterizing multiple types of tumors. The problem of multiple classifications has recently received more attention in the context of DNA microarrays. In the present study, we first presented an evaluation of the performance of LDA and its modification methods for classification with 6 public microarray datasets.

The gene selection method [6,24,25], the number of selected genes and the classification method are three critical issues for the performance of a sample classification. Feature selection techniques can be organized into three categories, filter methods, wrapper methods and embedded methods. LDA and its modification methods belong to wrapper methods which embed the model hypothesis search within the feature subset search. In the present study, different numbers of gene have been selected by different LDA modification methods. There is no theoretical estimation of the optimal number of selected genes and the optimal gene set can vary from data to data [26]. So we did not focus on the combination of the optimal gene set by one feature gene selection method and one classification algorithm. In this paper we just describe the performance of LDA and its modification methods under the same selection method in different microarray dataset.

Various statistical and machine learning methods have been used to analyze the high dimensional data for cancer classification. These methods have been shown to have statistical and clinical relevance in cancer detection for a variety of tumor types. In this study, it has been shown that LDA modification methods have better performance than traditional LDA under the same gene selection criterion. Dudoit also reported that simple classifiers such as DLDA and Nearest Neighbor performed remarkably well compared with more sophisticated ones, such as aggregated classification trees [27]. It indicates that LDA modification methods did a good job in some situations. Zhang *et al *[28] developed a fast algorithm of generalized linear discriminant analysis (GLDA) and applied it to seven public cancer datasets. Their study included 4 same datasets (Colon, Prostate, SRBCT and Brain) as those in our study and adopted a 3-fold cross-validation design. The average test errors of our study were less than those of their study, while there was no statistical significance of the difference. The results reported by Guo *et al *[4] are of concordance with ours except for the colon dataset. Their study also included the above mentioned 4 same datasets and they found that in the colon dataset the average test error of SCRDA was as same as PAM, while in the present study we found that the average test error of SCRDA was slightly less than that of PAM.

There are several interesting problems that remain to be addressed. A question is raised that when comparing the predictive performance of different classification methods on different microarray data, is there any difference between various methods, such as leave-one-out cross-validation and bootstrap [29,30]? And another interesting further step might be a pre-analysis of the data to choose a suitable gene selection method. Despite the great promise of discriminant analysis in the field of microarray technology, the complexity and the multiple choices of the available methods are quite difficult to the bench clinicians. This may influence the clinicians' adoption of microarray data based results when making decision on diagnosis or treatment. Microarray data's widespread clinical relevance and applicability still need to be resolved.

## Conclusions

An extensive survey in building classification models from microarray data with LDA and its modification methods has been conducted in the present study. The study showed that the modification methods are superior to LDA in the prediction accuracy.

## List of abbreviations

CV: Cross-validation; DDA: diagonal discriminant analysis; FNDR: False non-discovery rates; GLDA: generalized linear discriminant analysis; HCT: Higher criticism threshold; LDA: linear discriminant analysis; NSC: nearest shrunken centroid method; PAM: prediction analysis for microarrays; SCRDA: Shrinkage centroid regularized discriminant analysis; SDA: Shrinkage discriminant analysis; SDDA: Shrinkage diagonal discriminant analysis; SLDA: Shrinkage linear discriminant analysis.

## Competing interests

The authors declare that they have no competing interests.

## Authors' contributions

DH conceived the study and drafted the manuscript. DH and YQ performed the analyses. MH provided guidance and discussion on the methodology. BZ attracted partial funding and participated in the design of the analysis strategy. All authors read and approved the final version of this manuscript.
